# Beyond Adrenal Crisis: Protean Manifestations of Hypocalcemia in Autoimmune Polyglandular Syndrome Type 1

**DOI:** 10.1016/j.aed.2025.12.014

**Published:** 2025-12-30

**Authors:** Bryan B. Franco, Sola Mansour, Keysun Ranjbar, Donald Morrish, Peter M. Hwang

**Affiliations:** 1 Division of General Internal Medicine, University of Alberta, Edmonton, Alberta; 2 Division of Endocrinology, University of Alberta, Edmonton, Alberta; 3 Division of Cardiology, University of Alberta, Edmonton, Alberta

**Keywords:** adrenal insufficiency, APS type 1, cardiomyopathy, hypocalcemia, urinary retention

## Abstract

**Introduction:**

Autoimmune polyglandular syndrome type 1 (APS1) is associated with adrenal insufficiency, hypoparathyroidism, mucocutaneous candidiasis, and primary ovarian insufficiency. Our objective is to describe 2 acute presentations unrelated to adrenal crisis in a patient with APS1: 1) stress-induced cardiomyopathy presenting as shock and heart failure and 2) acute on chronic urinary retention.

**Case report:**

A 40-year-old patient with APS1 was admitted to the intensive care unit with cardiogenic shock. She was diagnosed with nonischemic cardiomyopathy with a left ventricular ejection fraction of 20% to 25% in the setting of severe hypocalcemia. Her heart function recovered after 1 year, and the working diagnosis was stress-induced cardiomyopathy complicated by hypocalcemia.

At 50 years old, she presented to the emergency department with abdominal pain and tingling in her hands and feet. She was found to have severe hypocalcemia as well as acute on chronic urinary retention. After intravenous calcium supplementation, her symptoms subsided, and she was able to void once again and was discharged home without urinary catheter. Subsequent urological studies demonstrated detrusor overactivity and severe pelvic floor dysfunction.

**Discussion:**

Acute hypocalcemia is known to cause neuromuscular irritability, and there is increasing evidence that this can also precipitate stress-induced cardiomyopathy and urinary retention. Female APS1 patients are further predisposed to both conditions due to hypoestrogenism from primary ovarian failure.

**Conclusion:**

Atypical sequelae of hypoparathyroidism-associated hypocalcemia, such as cardiomyopathy and urinary retention, warrant consideration in acute presentations of patients with APS1.

**Highlights**

• Autoimmune polyglandular syndrome type 1 (APS1) causes hypoparathyroidism
• Hypocalcemia can result in presentations such as heart failure and urinary retention
• In APS1, sequelae of hypocalcemia should be considered in acute presentations

**Clinical Relevance**

While adrenal crisis is a feared complication of patients with autoimmune polyglandular syndrome type 1 presenting acutely, sequelae related to hypoparathyroidism-related hypocalcemia can take many acute forms and should prompt rapid correction of hypocalcemia and diligent maintenance of normal calcium levels through supplementation of calcium and vitamin D.

## Introduction

Autoimmune polyglandular syndrome type 1 (APS1) is a rare monogenic disease arising from mutations in the autoimmune regulator gene, leading to organ damage, particularly in endocrine glands, caused by abnormal clones of immune cells.^1^ APS1 presents as a classic triad of mucocutaneous candidiasis, adrenal insufficiency, and hypoparathyroidism in childhood.^1^^,^^2^ Other features can include vitiligo, asplenia, pneumonitis, hypogonadism, diabetes, hypothyroidism, and hepatitis.^2^

Adrenal crisis is a feared complication of APS1 and is frequently managed empirically when patients present acutely to care. In this report, we describe 2 separate acute presentations that were initially attributed to adrenal crisis, though this was later found to be noncontributory. Upon closer analysis, the patient was found to be markedly hypocalcemic during both episodes. Hypocalcemia is known to cause neuromuscular irritability by decreasing the neuronal threshold of activation via calcium-regulated ion channels. Although hypocalcemia is generally asymptomatic, certain manifestations can be provoked in susceptible individuals, such as seizures, paresthesias, muscle spasm (Chvostek’s sign and Trousseau’s sign), bronchospasm, and diarrhea.^3^ These could be seen as a decrease in the activation threshold of neurons in the central and peripheral nervous systems, with downstream effects on skeletal and smooth muscle.

We present a patient with APS admitted on 2 separate occasions with 2 unusual manifestations of hypocalcemia: 1) stress-induced cardiomyopathy presenting as shock and heart failure, and 2) acute urinary retention.

## Case Report

### Cardiomyopathy

A 40-year-old woman presented to the emergency department with abdominal pain and feeling unwell. Four weeks prior, she was involved in a motor vehicle accident without suffering serious injuries. She was hypotensive on arrival and required intensive care unit admission for 2 weeks for shock requiring support with vasoactive medications and heart failure requiring endotracheal intubation. She was treated empirically for adrenal crisis with hydrocortisone intravenous (IV) given her history of adrenal insufficiency. Table summarizes relevant laboratory investigations at presentation.

**Table:** Key Laboratory Values at Presentation and Admission to the Intensive Care Unit

Laboratory	
Sodium	127 mmol/L (reference 133-146 mmol/L)
Potassium	5.2 mmol/L (reference 3.5-5.0 mmol/L)
Creatinine	185 umol/L (prior available Cr was 78 umol/L)
Glucose	2.4 mmol/L (reference 3.3-11.0 mmol/L)
Calcium	1.89 mmol/L (reference 2.10-2.60 mmol/L), nadir 1.25 mmol/L on Day 10 of her admission.
Random cortisol	2901 nmol/L (reference 85-620 nmol/L)
Troponin I	0.19 μg/L (reference <0.15ug/L), peaked at 4.02 μg/L on Day 3 of admission.

The patient had been diagnosed with APS1 at around 10 years old. This manifested as adrenal insufficiency, chronic mucocutaneous candidiasis, primary hypoparathyroidism (PTH 4 ng/L, reference 15-65 ng/L), primary ovarian insufficiency (LH 44.8 U/L, FSH 49 U/L, estradiol <40 pmol/L at 39 years old), pernicious anemia, and vitiligo. Her thyroid function was normal (TSH 1.78 mU/L, reference 0.20-4.00 mU/L). She was prescribed long-term hydrocortisone, calcium, calcitriol, vitamin D, estrogen, progesterone, and vitamin B12. She has had longstanding issues with medication coverage, leading to occasional lapses in medication adherence.

Random cortisol was elevated on admission (Table). Investigations for other causes of shock, including an infectious work-up, were negative. While her initial chest x-ray did not demonstrate any abnormalities, she subsequently developed bilateral pleural effusions.

Her initial electrocardiogram shown in Figure, revealed a prolonged corrected QT interval of 494 ms. Transthoracic echocardiography showed a left ventricular ejection fraction (LVEF) of 20% to 25%, with akinesis of the septum and apex. She had elevated troponin, but there was no significant obstructive coronary artery disease on angiogram. She improved clinically in the intensive care unit with management of heart failure and hypocalcemia (requiring multiple doses of IV and oral repletion). At discharge, her corrected QT interval was 454 ms. She was discharged with additional medications: bisoprolol, ramipril, spironolactone, and furosemide.

**Fig:**
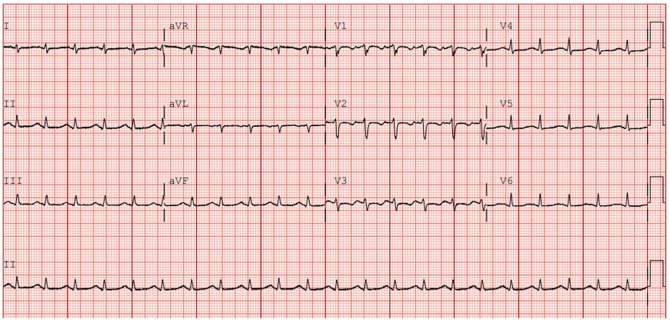
Presenting electrocardiogram. Sinus tachycardia with corrected QT interval 494 ms.

Cardiac MRI 6 months after admission showed an LVEF of 46% and only mild residual wall motion abnormalities. One year later, her cardiac function recovered further, with subsequent transthoracic echocardiograms demonstrating an LVEF of 55% without any regional wall motion abnormalities. She has not had any further issues with heart failure.

### Urinary Retention

At the age of 50, the same patient presented to the emergency department with acute worsening of pre-existing urinary retention along with abdominal pain and tingling in her hands and feet. She was found to have severe hypocalcaemia, with a serum calcium of 1.59 mmol/L (reference 2.10-2.60 mmol/L). Notably, she had stopped her home calcium, calcitriol, and vitamin D due to lack of funds.

Given her history, she was treated with hydrocortisone IV empirically for adrenal crisis and provided antibiotics for possible urinary tract infection, which was stopped when her urinalysis did not show pyuria. Calcium was supplemented intravenously and orally, and at discharge, her serum calcium improved to 2.17 mmol/L (reference 2.10-2.60). Following calcium supplementation, she regained the ability to void without catheterization, and urology follow-up was arranged as an outpatient.

The patient first reported intermittent symptoms of urinary retention at the age of 37. Retention was confirmed with ultrasound imaging (750 pre-void bladder volume and 700 post-void residual). She underwent urodynamic studies that showed detrusor overactivity at that time. A Foley catheter was inserted, but fortunately her symptoms subsided, and she was able to void again after its removal. A similar episode recurred at age 50, as described above. This time, during cystoscopy, she was noted to have a very active pelvic floor and was diagnosed with severe pelvic floor dysfunction. She was referred for pelvic physiotherapy and has not required repeat urinary catheterization. Notably, the patient has never been pregnant, owing to her primary ovarian insufficiency.

## Discussion

This case outlines an adult patient with APS1 with 2 different acute presentations brought on by hypocalcemia of hypoparathyroidism, predisposed by pre-existing abnormalities.

Takotsubo cardiomyopathy, or stress-induced cardiomyopathy, is most common in postmenopausal women and can be precipitated by severe psychological, emotional, or physical stress as well as stimulants or related medications.^4^ It is believed to be caused by sympathetic-mediated vasoconstriction of small blood vessels, giving rise to ischemic cardiac muscle injury that can resemble type 1 myocardial infarction due to acute thrombotic occlusion of a major artery. However, it presents with a different pattern of wall motion abnormalities corresponding to watershed areas of the vascular supply, classically at the apex in Takotsubo cardiomyopathy. The damage is usually reversible, unlike the permanent scarring that occurs in infarct due to type 1 MI. Our patient was susceptible to stress-induced cardiomyopathy due to psychological and emotional stress from her recent car accident, as well as having gone through early menopause from primary ovarian failure. Severe hypocalcemia likely also contributed, as there have been many case reports in which hypocalcemia was thought to be the primary inciting factor in Takotsubo or stress-induced cardiomyopathy.^5^, ^6^, ^7^, ^8^, ^9^ In addition, hypocalcemia and cardiomyopathy have been reported in a pediatric case of APS1.^10^

Acute on chronic urinary retention from pelvic floor dysfunction is not a typical manifestation of APS1 described in the literature. However, primary ovarian insufficiency may have predisposed our patient to pelvic floor dysfunction, which can manifest as chronic pelvic pain, sexual problems, lower urinary tract symptoms, or as in our patient’s case, dysfunctional voiding.^11^ Pelvic floor dysfunction is often associated with multiple vaginal deliveries, but in our patient, it may have been related to hypoestrogenism. Hypoestrogenism in menopause is thought to cause alterations in connective tissue, resulting in weakened ligament support and pelvic floor dysfunction.^11^ While the exact mechanism is unclear, our patient was known to be prone to urinary retention, and this was exacerbated in the setting of hypocalcemia due to nonadherence to calcium and vitamin D therapy. Acute hypocalcemia due to hypoparathyroidism was recently found to be associated with bladder dysfunction and elevated post-void residuals.^12^

## Conclusions

While adrenal crisis is a feared complication of APS1 and is often treated empirically when patients present acutely, there are several potential sequelae of hypoparathyroidism-associated hypocalcemia that should be considered. Our patient had 2 separate hospital admissions related to hypocalcemia, though she and many of the initial treating physicians were aware only of adrenal insufficiency-related complications of APS1. Our patient was already aware of the importance of steroid replacement therapy and stress dosing, but after her admission she was also educated about the importance of vitamin D and calcium supplementation for APS1.

## Patient Consent

The patient provided written informed consent for this case report.

## Disclosure

The authors have no conflicts of interest to disclose.
